# A Multicenter Evaluation of Vancomycin-Associated Acute Kidney Injury in Hospitalized Patients with Acute Bacterial Skin and Skin Structure Infections

**DOI:** 10.1007/s40121-019-00278-1

**Published:** 2020-01-25

**Authors:** Sarah C. J. Jorgensen, Kyle P. Murray, Abdalhamid M. Lagnf, Sarah Melvin, Sahil Bhatia, Muhammad-Daniayl Shamim, Jordan R. Smith, Karrine D. Brade, Samuel P. Simon, Jerod Nagel, Karen S. Williams, Jessica K. Ortwine, Michael P. Veve, James Truong, David B. Huang, Susan L. Davis, Michael J. Rybak

**Affiliations:** 1 Eugene Applebaum College of Pharmacy & Health Sciences, Wayne State University, Detroit, MI USA; 2 Detroit Medical Center, Detroit, MI USA; 3 Fred Wilson School of Pharmacy, High Point University, High Point, NC USA; 4 Cone Health, Greensboro, NC USA; 5 Boston Medical Center, Boston, MA USA; 6 Maimonides Medical Center, Brooklyn, NY USA; 7 University of Michigan, Ann Arbor, MI USA; 8 Guthrie Robert Packer Hospital, Sayre, PA USA; 9 Parkland Memorial Hospital, Dallas, TX USA; 10 College of Pharmacy, University of Tennessee Health Sciences Center, Knoxville, TN USA; 11 University of Tennessee Medical Center, Knoxville, TN USA; 12 The Brooklyn Hospital Center, Brooklyn, NY USA; 13 Motif BioSciences, Princeton, NJ USA; 14 Rutgers New Jersey Medical School, Trenton, NJ USA; 15 Henry Ford Health-System, Detroit, MI USA; 16 School of Medicine, Wayne State University, Detroit, MI USA

**Keywords:** Acute bacterial skin and soft tissue infection, Acute kidney injury, Nephrotoxicity, Vancomycin

## Abstract

**Background:**

We sought to determine the real-world incidence of and risk factors for vancomycin-associated acute kidney injury (V-AKI) in hospitalized adults with acute bacterial skin and skin structure infections (ABSSSI).

**Methods:**

Retrospective, observational, cohort study at ten U.S. medical centers between 2015 and 2019. Hospitalized patients treated with vancomycin (≥ 72 h) for ABSSSI and ≥ one baseline AKI risk factor were eligible. Patients with end-stage kidney disease, on renal replacement therapy or AKI at baseline, were excluded. The primary outcome was V-AKI by the vancomycin guidelines criteria.

**Results:**

In total, 415 patients were included. V-AKI occurred in 39 (9.4%) patients. Independent risk factors for V-AKI were: chronic alcohol abuse (aOR 4.710, 95% CI 1.929–11.499), no medical insurance (aOR 3.451, 95% CI 1.310–9.090), ICU residence (aOR 4.398, 95% CI 1.676–11.541), Gram-negative coverage (aOR 2.926, 95% CI 1.158–7.392) and vancomycin duration (aOR 1.143, 95% CI 1.037–1.260). Based on infection severity and comorbidities, 34.7% of patients were candidates for oral antibiotics at baseline and 39.3% had non-purulent cellulitis which could have been more appropriately treated with a beta-lactam. Patients with V-AKI had significantly longer hospital lengths of stay (9 vs. 6 days, *p* = 0.001), higher 30-day readmission rates (30.8 vs. 9.0%, *p* < 0.001) and increased all-cause 30-day mortality (5.1 vs. 0.3%, *p* = 0.024)

**Conclusions:**

V-AKI occurred in approximately one in ten ABSSSI patients and may be largely prevented by preferential use of oral antibiotics whenever possible, using beta-lactams for non-purulent cellulitis and limiting durations of vancomycin therapy.

**Electronic supplementary material:**

The online version of this article (10.1007/s40121-019-00278-1) contains supplementary material, which is available to authorized users.

## Key Summary Points

**Table:** 

In patients with at least one baseline risk factor for acute kidney injury (AKI) treated with vancomycin for acute bacterial skin and skin structure infections, vancomycin-associated acute kidney injury (V-AKI) occurred in 9.4%.
Each additional day of vancomycin therapy beyond day 3 increased the odds of V-AKI by 14.3%.
The majority of patients in this study could have been more appropriately treated with safer alternatives.
V-AKI may be largely prevented by preferential use of oral antibiotics whenever possible, using beta-lactams for non-purulent cellulitis and limiting durations of vancomycin therapy.

## Introduction

Acute bacterial skin and skin structure infections (ABSSSIs) are the most common infections encountered in both the ambulatory and inpatient settings [1–4]. In 2013, approximately 3.4 million individuals sought emergency department care for ABSSSI in the United States [1]. Although rates have recently stabilized, this figure represents a three-fold increase over two decades earlier and has coincided with rising hospital admission rates, secondary complications and healthcare resource utilization [1, 5–7]. The increases in ABSSSI incidence and severity have been linked to the emergence, spread and persistence of community-acquired methicillin-resistant *Staphylococcus aureus* (CA-MRSA) as a predominant pathogen [7]. Although the prevalence of invasive MRSA infections may be declining in some regions, it remains a predominant pathogen among patients with purulent ABSSSI in the US [8–11].

Vancomycin has been the standard therapy for serious ABSSSI caused by resistant Gram-positive bacteria for decades [9, 12]. It remains the antibiotic most extensively studied in ABSSSI randomized controlled clinical trials (RCTs) with efficacy and safety outcomes generally similar to new comparators [13, 14]. Guidelines recommend vancomycin for first-line treatment of ABSSSI due to suspected or confirmed MRSA with the highest strength recommendation [9, 15]. Despite this support and its widespread use, recognition of vancomycin’s shortcomings [poor tissue penetration, decreased activity against strains with higher minimum inhibitory concentrations (MICs) or the heteroresistant phenotype, need for therapeutic drug-monitoring and nephrotoxicity] has led many experts to question its future role in the treatment of ABSSSI [13, 16, 17]. To counter concerns about reduced effectiveness, experts have recommended more intensive vancomycin dosing in serious infections, targeting serum trough concentration levels between 15 and 20 mg/L as a surrogate for area under the concentration–time curve (AUC) ≥ 400 [18, 19]. More aggressive vancomycin dosing has been associated with increased reports of vancomycin-associated acute kidney injury (V-AKI) [19, 20]. On the other hand, less intensive vancomycin dosing is recommended for non-severe ABSSSI [15], and these patients may be candidates for early transition to oral therapy, attenuating vancomycin exposure-associated renal injury. Furthermore, RCTs comparing vancomycin to other anti-MRSA agents for the treatment of ABSSSI have documented low overall rates of renal adverse events [13, 14]. There are a number of reasons to question whether the low rates of V-AKI observed in ABSSSI RCTs can be generalized to the real-world setting. First, patients with well-described risk factors for V-AKI were either excluded or underrepresented in these studies [13]. Antibiotics are administered for a pre-specified duration, generally 7–14 days, and in some RCTs there is the option to transition to oral therapy around day 3 [21–24]. In the real-world setting, patients have multiple risk factors, are frequently on concomitant nephrotoxic medications, and intravenous (IV) antibiotics are often continued long after patients became candidates for oral therapy [6, 19, 25, 26]. This is concerning, because some studies have shown that the risk of V-AKI increases with longer durations of therapy [19]. Finally, although a clear distinction can be made between a patient presenting with necrotizing fasciitis and a stable patient with an uncomplicated abscess, the vast majority of ABSSSIs fall within a gray area between these entities, and clinicians may err on the side of caution with a more intensive vancomycin dosing schedule.

V-AKI is typically reversible; however, AKI can further complicate a patient’s hospital course and has been associated with prolonged hospital and intensive care unit (ICU) lengths of stay and increased overall mortality [19, 27–29]. Given the large number of patients treated with vancomycin for ABSSSI, V-AKI in this group would be expected to exert a tremendous burden on the healthcare system. Recent studies have demonstrated reduced rates of V-AKI when vancomycin is dosed to target an AUC/MIC of 400–600, rather than trough-based dosing [30, 31]. Perceived challenges with implementation of AUC-based monitoring (time allocation, training requirements, costs) may dissuade its use in non-critically ill ABSSSI patients. Newer agents with proven efficacy for MRSA infections are also available, but are not routinely prescribed for ABSSSI because of higher drug acquisition costs and/or relative lack of experience compared to vancomycin.

These considerations underscore the need to better characterize V-AKI with real-world use among hospitalized patients treated with this agent for ABSSSI. The objectives of this study were therefore to (1) determine the incidence of V-AKI with real-world use among hospitalized patients with ABSSSI, (2) identify baseline and treatment-related risk factors for V-AKI among hospitalized ABSSSI patients, and (3) explore antimicrobial stewardship opportunities to improve appropriate vancomycin use in ABSSSIs.

## Study Design

This was a multicenter, retrospective, observational, cohort study conducted from January 2015 to June 2019 at ten US medical centers (seven academic urban and three academic rural) representative of a diverse geographic region and patient population. Approval was obtained from each center’s institutional review board (IRB) with a waiver for informed consent. The Wayne State University IRB served as the coordinating IRB. Potential patients were screened from reports generated based on International Classification of Diseases, 10th Revision, Clinical Modification (ICD-10-CM) diagnostic codes for ABSSSI. Patients were eligible for the study if they met the following criteria: (1) age ≥ 18 years; (2) IV vancomycin initiated ≤ 24 h of admission and continued for ≥ 72 h; (3) ABSSSI per the US Food and Drug Administration definition [32]; and (4) ≥ one baseline risk factor for AKI (Supplementary Appendix 1) [27, 33–37]. Patients were excluded if any of the following were true: (1) baseline serum creatinine > 4.0 mg/dL; (2) end-stage renal disease or receipt of chronic or emergent renal replacement therapy prior to vancomycin initiation; (3) AKI at the time of vancomycin initiation [38]; (4) concomitant infection requiring systemic antimicrobial therapy when vancomycin was initiated (patients with bacteremia thought to be from an ABSSSI source were included); or (5) pregnant or nursing mother. Relevant patient demographic, clinical, treatment and outcome data were extracted from the electronic medical record by study investigators at each center and entered into a secured electronic data collection form [39]. Patients were followed until hospital discharge. Readmissions to the same institution within 30 days of discharge were also recorded. A convenience sample of 500 patients was targeted based on feasibility and anticipated funding including 200 patients from the lead investigator’s site and 30 patients each from ten additional medical centers. Due to early study termination, the target sample size was not met.

Comorbidity burden was quantified by calculating the Charlson Comorbidity score [40]. Immunosuppression was defined as previously described [41]. The systemic inflammatory response syndrome (SIRS), quick Sequential Organ Failure Assessment (qSOFA) and severe sepsis were evaluated according to consensus guidelines using the worst physiological measurements recorded within 24 h of vancomycin initiation [37, 42]. The Dundee classification was used to determine severity of illness at infection onset [41, 43]. The Dundee classification is a validated scoring system for ABSSSI based on routinely measured objective physiological parameters and the presence of comorbidities that may complicate or delay the resolution of infection [41, 43]. Patients in class I are candidates for oral antibiotic therapy targeted to *Streptococcus pyogenes* and *S. aureus*. Initial IV antibiotic therapy, targeted to these Gram-positive pathogens is appropriate for patients in classes II and III, while therapy should be broadened to cover Gram-negative and anaerobic bacteria for class IV [41, 43]. Initial serum creatinine was measured within 24 h of the first vancomycin dose. Peak serum creatinine was the highest value recorded while receiving vancomycin to within 72 h of discontinuation. Glomerular filtration rate (GFR) was estimated using the Cockcroft–Gault Modification of Diet and Renal Disease (MDRD) and Chronic Kidney Disease Epidemiology Collaboration (CKD-EPI) equations [36, 44, 45] Where available, vancomycin exposure was evaluated using initial steady-state trough concentrations [19]. Vancomycin was dosed and monitored according to each hospital’s standard of care.

The primary outcome was V-AKI evaluated according to vancomycin guidelines definition as an increase from baseline in serum creatinine of ≥ 0.5 mg/L or 50% on ≥ two consecutive measurements [18]. V-AKI was also evaluated according to the RIFLE (risk, injury, failure, loss, end-stage renal disease) and the Acute Kidney Injury Network (AKIN) criteria. [46, 47] The risk period was from vancomycin initiation to 72 h after the last dose or at hospital discharge, which ever occurred first [48].

### Statistical Analysis

Descriptive statistics were used to characterize the cohort. Continuous variables were reported as medians (interquartile ranges, IQR), whereas categorical variables were expressed as counts and percentages. Bivariate associations between covariates and V-AKI were expressed as odds ratios and 95% confidence intervals (CIs).

To identify independent predictors of V-AKI, we modeled both occurrence of V-AKI and time to V-AKI. Occurrence of V-AKI was evaluated by multivariable logistic regression. Covariates with a* p *value less than 0.2 and with biological plausibility by unadjusted analyses were entered into the model through defined blocks starting with baseline demographic and clinical variables followed by vancomycin-related variables and other management-related variables. Stepwise techniques were used to build a parsimonious model with variables remaining in the final model if the adjusted *p* value was < 0.05. Multi-collinearity was assessed via the variance inflation factor, with values < 3 being considered acceptable. Effect modification was also evaluated. To examine time to V-AKI, we employed Cox proportional hazards regression. Patients who did not experience V-AKI were censored 72 h after the last dose of vancomycin or at hospital discharge, which ever occurred first.

Two sensitivity analyses were performed: (1) excluding cases enrolled from the Detroit Medical Center which was our largest contributor, and (2) excluding sites that contributed less than ten cases.

All calculations were performed with SAS 9.4 Statistical Software (SAS Institute, Cary, NC, USA) and SPSS, v.25 (IBM. Armonk, NY, USA).

## Results

A total of 415 patients met inclusion criteria and were evaluated, representing 57,143 vancomycin-days. Demographic, clinical and infection characteristics are shown in Tables 1 and 2. The median age was 58 (46–69) years and approximately half (52.3%) were male. Both Caucasian and African American patients were well represented (46.3% and 42.7%, respectively). The majority of patients (55.4%) were obese. Diabetes mellitus (36.6%), chronic obstructive pulmonary disease (16.9%) and psychiatric illnesses (15.7%) were the most common comorbidities. The median GFR at initial evaluation ranged from 94 (71–129) mL/min/1.73 m^2^ (MDRD) to 98 (71–129) mL/min (Cockroft–Gault) with few patients (4.6–7.5%) having a GFR under 45 mL/min. Nearly half (48.7%) of patients meet criteria for sepsis but few patients (3.1%) had severe sepsis at initial evaluation. With regards to the Dundee classification for infection severity, 144 (34.7%) were in severity class I, 69 (16.6%) in class II, 136 (32.8%) in class III and 66 (15.9%) in class IV. The most common ABSSSI type and location were non-purulent cellulitis (39.3%) and lower extremity (59.8%), respectively.

**Table 1:** Baseline patient and hospital characteristics and unadjusted odds ratios for vancomycin-associated acute kidney injury

Characteristic	*n* = 415 *n* (%) or median (IQR)	Odds ratio (95% confidence interval)	*p* value
Age, years	58 (46–69)	0.996 (0.976–1.016)	0.671
Age ≥ 65 years	150 (36.1)	0.988 (0.497–1.965)	0.973
Sex			
Male	217 (52.3)	1.711 (0.863–3.394)	0.121
Female	198 (47.7)	Reference	–
Actual body weight (kg)	91 (74–115)	1.004 (0.996–1.011)	0.323
Body mass index (kg/m^2^)	31 (25–39)	1.010 (0.986–1.034)	0.416
Obese (BMI ≥ 30 kg/m^2^)	230 (55.4)	0.932 (0.481–1.807)	0.835
Baseline serum creatinine (mg/dL)	0.82 (0.70–1.00)	1.386 (0.521–3.685)	0.513
Baseline serum creatinine > 1.5 mg/dL	13 (3.1)	0.798 (0.101–6.308)	1.000
Baseline estimated GFR			
Cockroft Gault (mL/min)	98 (71–129)	1.002 (0.996–1.009)	0.486
CG < 45 mL/min/1.73 m^2^	31 (7.5)	0.647 (0.148–2.920)	0.559
MDRD (mL/min/1.73 m^2^)	94 (74–114)	1.000 (0.990–1.009)	0.943
MDRD < 45 mL/min/1.73 m^2^	19 (4.6)	1.141 (0.254–5.135)	0.863
CKD-EPI (mL/min/1.73 m^2^)	97 (78–117)	0.998 (0.986–1.009)	0.687
CKD-EPI < 45 mL/min/1.73 m^2^	223 (5.5)	0.423 (0.056–3.230)	0.393
Race			
Caucasian	192 (46.3)	Reference	–
African American	177 (42.7)	0.440 (0.203–0.953)	0.037
Other^a^	46 (11.1)	1.102 (0.412–2.885)	0.843
Hospital			
A	193 (46.5)	Reference	–
B	58 (14)	1.026 (0.321–3.276)	0.966
C	30 (7.2)	2.769 (0.910–8.428)	0.073
D	2 (0.5)	0	1.0
E	30 (7.2)	0.477 (0.060–3.789)	0.484
F	4 (1.0)	0	1.0
G	30 (7.2)	0.989 (0.212–4.618)	0.989
H	16 (3.9)	4.615 (1.304–16.334)	0.018
I	22 (5.3)	2.186 (0.572–8.361)	0.253
J	30 (7.2)	1.538 (0.411–5.753)	0.522
Medical insurance			
Medicare	137 (33.0)	Reference	–
Medicaid	68 (16.4)	0.827 (0.279–2.450)	0.731
Private	129 (31.1)	0.875 (0.365–2.102)	0.766
Mixed	49 (11.8)	0.926 (0.284–3.019)	0.898
Uninsured	32 (7.7)	3.472 (1.283–9.396)	0.014
Admission source			
Home	375 (90.4)	Reference	–
Nursing facility	27 (6.5)	0.753 (0.171–3.312)	0.708
Homeless	8 (1.9)	1.345 (0.161–11.244)	0.784
Other	5 (1.2)	0	1.00
Social history			
Tobacco use	101 (24.3)	1.433 (0.697–2.946)	0.325
Alcohol abuse	39 (9.4)	4.126 (1.831–9.298)	< 0.001
IV drug abuse	38 (9.2)	1.529 (0.560–4.173)	0.405
Marijuana use	35 (8.4)	0.896 (0.261–3.072)	0.861
Comorbidities			
Heart failure	61 (14.7)	1.573 (0.686–3.606)	0.281
Diabetes mellitus	152 (36.6)	1.736 (0.895–3.367)	0.100
COPD	70 (16.9)	1.816 (0.841–3.922)	0.124
Peripheral vascular disease	42 (10.1)	1.717 (0.674–4.375)	0.257
Chronic venous insufficiency	16 (3.9)	0.633 (0.081–4.928)	0.663
Cancer	48 (11.6)	0.862 (0.292–2.542)	0.788
Liver disease	24 (5.8)	0.404 (0.053–3.075)	0.366
Immunosuppression	22 (5.3)	0.445 (0.058–3.400)	0.423
Psychiatric disorder	65 (15.7)	1.714 (0.772–3.804)	0.181
Charlson Comorbidity score	1 (0–3)	1.141 (0.986–1.322)	0.077
Hospitalization within 90 days	92 (22.2)	1.880 (0.924–3.825	0.078

**Table 2:** Baseline infection and illness severity characteristics and unadjusted odds ratios for vancomycin-associated acute kidney injury

Characteristic	*n* = 415 *n* (%) or median (IQR)	Odds ratio (95% confidence interval)	*p* value
Dundee class			
Class 1	144 (34.7)	Reference	–
Class 2	69 (16.6)	0.529 (0.169–1.659)	0.275
Class 3	136 (32.8)	0.909 (0.416–1.988)	0.811
Class 4	66 (15.9)	1.020 (0.395–2.635)	0.967
ABSSSI type			
Non-purulent cellulitis	163 (39.3)	Reference	–
Purulent cellulitis	99 (23.9)	1.296 (0.546–3.079)	0.556
Cutaneous abscess	92 (22.2)	1.099 (0.438–2.758)	0.841
Wound infection	61 (14.7)	1.742 (0.684–4.435)	0.245
ABSSSI location			
Upper extremity	60 (14.5)	Reference	
Lower extremity	248 (59.8)	1.100 (0.432–2.796)	0.842
Torso	57 (13.7)	0.327 (0.063–1.694)	0.183
Buttock/perianal	24 (5.8)	0.818 (0.153–4.370)	0.814
Head/neck	26 (6.3)	0.750 (0.141–3.988)	0.736
Blood culture obtained	293 (70.6)	2.014 (0.864–4.697)	0.099
Positive blood culture	20 (4.8)	1.075 (0.240–4.816)	0.925
Failed outpatient therapy for index ABSSSI	94 (22.4)	1.195 (0.559–2.551)	0.646
Abnormal temperature (> 38 C or < 36 C)	96 (23.1)	1.163 (0.545–2.481)	0.696
Respiratory rate ≥ 22 breath/minute	76 (18.3)	0.795 (0.321–1.970)	0.619
Heart rate > 90 beats/minute	262 (63.1)	1.350 (0.663–2.750)	0.407
Systolic blood pressure ≤ 100 mmHg	87 (21.0)	0.970 (0.429–2.194)	0.942
Abnormal WBC (> 12 × 10^3^ or < 4 x 10^3^)	200 (48.2)	0.815 (0.419–1.584)	0.546
Altered mental status (GCS < 15)	20 (4.8)	2.571(0.815–8.115)	0.096
qSOFA ≥ 2	41 (9.9)	1.389 (0.511–3.774)	0.518
≥ 2 SIRS criteria present	202 (48.7)	1.122 (0.580–2.170)	0.732
Severe sepsis/septic shock	13 (3.1)	1.794 (0.383–8.401)	0.452
ICU admission within 24 h of hospital admission	30 (7.2)	4.152 (1.708–10.097)	0.001
Mechanical ventilation within 24 h of admission	7 (1.7)	4.011 (0.752–21.397)	0.080
Skin/tissue/fluid specimen obtained^b^	195 (77.4)	0.348 (0.150–0.809)	0.014
*Staphylococcus aureus*^b^	102 (40.5)		
MSSA^b^	33 (13.1)		
MRSA^b^	69 (27.4)		
*Streptococcus* spp.^b^	35 (13.9)		
Gram-negative^b^	30 (11.9)		
*Escherichia coli*	7 (2.8)		
*Klebsiella pneumonia*	6 (2.4)		
*Pseudomonas aeruginosa*	4 (1.6)		
No organisms/normal flora^b,c^	41 (16.3)		

*ABSSSI* acute bacterial skin and skin structure infection, *BMI* body mass index, *C* Celsius, *CG* Cockroft–Gault, *CKD-EPI* chronic kidney disease epidemiology collaboration, *CT* computed tomography, *GCS* Glasgow Coma Scale, *ICU* intensive care unit, *MDRD* Modification of Diet in Renal Disease, *MSSA* methicillin-sensitive *S. aureus*, *MRSA* methicillin-resistant *S. aureus*, *MRI* magnetic resonance imaging, *OR* operating room, *SIRS* systemic inflammatory response syndrome, *qSOFA* quick Sequential Organ Failure Assessment, *WBC* white blood cell count

^a^Latino n = 20, Asian n = 3, Unknown *n* = 23

^b^n = 252 excluding patients with non-purulent cellulitis from whom a culture could not be obtained

^c^Normal flora as per local laboratory definitions

ABSSSI management is summarized in Table 3. Among patients from whom a tissue or fluid specimen could potentially be obtained (i.e., purulent cellulitis, abscess and wound infection, *n* = 252), a culture was collected in 77.4*%. S. aureus* was the most frequently cultured bacterial species (MSSA 13.1% and MRSA 27.4%). Twelve percent of cultures were positive for Gram-negative bacteria. Overall, blood cultures were collected from 70.6% of patients. Blood cultures were obtained more frequently as Dundee class increased from I to IV (59.7%, 55.1%, 78.7%, 93.9%; *p* < 0.001 for trend). Approximately 5% of the cohort had secondary bacteremia (MSSA 1.2% and MRSA 2.9%). Infectious disease services were consulted in 57.8% of cases, radiological testing was performed in 57.8% and a source control procedure was performed in 41.9%. No interactions between these variables and Dundee class were observed.

**Table 3:** Management-related variables and unadjusted odds ratios for vancomycin-associated acute kidney injury

	*n* = 415 *n* (%) or median (IQR)	Odds ratio (95% confidence interval)	*P* value
Radiological test performed	240 (57.8)	1.514 (0.755–3.038)	0.240
X-ray	100 (24.1)		
CT-scan	101 (24.3)		
Ultrasound	66 (15.9)		
MRI	34 (8.2)		
Surgical consult	255 (61.4)	1.461 (0.718–2.973)	0.294
Dermatology consult	8 (1.9)	0.979 (0.964–0.993)	1.000
Infectious diseases consult	240 (57.8)	1.514 (0.755–3.038)	0.240
Source control procedure	174 (41.9)	0.960 (0.491–1.876)	0.905
Bedside incision and drainage	62 (14.9)		
OR incision and drainage	100 (24.1)		
Debridement	10 (2.4)		
Amputation	5 (1.2)		
Concomitant antipseudomonal antibiotic ≥ 72 h	178 (42.9)	2.603 (1.311–5.168)	0.006
Cefepime	84 (20.2)	1.407 (0.657–3.016)	0.380
Piperacillin-tazobactam	83 (20.0)	2.831 (1.411–5.681)	0.003
Receipt of IV contrast dye	97 (23.4)	2.241 (1.124–4.468)	0.019
Concomitant nephrotoxic drug ≥ 48 h	257 (61.9)	2.572 (1.151–5.748)	0.021
Piperacillin-tazobactam	86 (20.7)	2.685 (1.340–5.379)	0.005
Vasopressors	2 (0.5)	9.868 (0.605–160.960)	0.179
Loop diuretic	81 (19.5)	1.977 (0.954–4.097)	0.063
ACE-inhibitor	64 (15.4)	1.224 (0.515–2.908)	0.646
Angiotensin receptor blocker	25 (6.0)	0.830 (0.188–3.659)	1.000
Tenofovir disoproxil fumarate	4 (1.0)	3.272 (0.332–32.234)	0.327
Non-steroidal anti-inflammatory	79 (19.0)	0.600 (0.227–1.587)	0.299
Acyclovir	5 (1.2)	6.721 (1.088–41.513)	0.018

*ACE* angiotensin converting enzyme, *IV* intravenous; operating room

^a^*n* = 257

With regards to vancomycin therapy, the median daily dose was 26 (17–31) mg/kg. Daily doses > 4000 mg were uncommon (4.1%) and loading doses were used in 11.3%. The median duration of vancomycin was 5 (4–6) days. Vancomycin was administered for greater than 7 days to 20.7% of patients. In the subgroup of patients that had a skin specimen culture collected (*n* = 195), the length of vancomycin therapy [5 (4–6) vs. 5 (4–7), *p* = 0.888] and the proportion of patients that received > 7 days of vancomycin (18.8% vs 20.6%, *p* = 0.765), was similar in patients with and without an MRSA positive culture, respectively.

In total, 257 (61.9%) patients had at least one steady-state vancomycin trough measured; the median initial trough was 12.0 (8.4–16.0) mg/L and 68.5% of patients had an initial trough < 15 mg/L. Concomitant antibiotics with Gram-negative activity and/or antipseudomonal activity were used for $\ge$ 72 h in 59.8% and 42.9% of patients, respectively. The use of concomitant nephrotoxic medicines was also common (61.9%) and 23.4% of patients received parenteral contrast dye.

V-AKI according to the vancomycin consensus guidelines, the RIFLE and AKIN criteria, developed in 39 (9.4%), 50 (12.0%) and 64 (15.4%) patients, respectively (Table 4). The median time to V-AKI onset was 4 (3–6) days. Amongst patients who experienced V-AKI, vancomycin was discontinued due to V-AKI in 26 (61.5%). V-AKI resolution occurred before or at the time of discharge in 26 (61.5%) patients. Resolution was not associated with vancomycin discontinuation; resolution occurred in 65.4% who continued vancomycin compared to 53.8% who discontinued vancomycin due to V-AKI (*p* = 0.485). Nephrology was consulted in 13 (3.1%) patients with V-AKI and emergent hemodialysis was initiated in 4 (1.0%).

**Table 4:** Vancomycin-associated acute kidney injury

Definition	*n* = 415 *n* (%)
Consensus Guidelines definition	39 (9.4)
RIFLE	50 (12.0)
Risk	32 (7.7)
Injury	10 (2.4)
Failure	8 (1.9)
AKIN	64 (15.4)
Stage 1	47 (11.3)
Stage 2	10 (2.4)
Stage 3	7 (1.7)

*AKIN* acute kidney injury network, *RIFLE* risk, injury, failure, loss of function, end-stage renal diseases

Bivariate associations between baseline demographic, clinical and infection variables and V-AKI are shown in Tables 1 and 2. No medical insurance, chronic alcohol abuse and ICU admission within 24 h of vancomycin initiation were positively associated with V-AKI, while African American race was protective (*p* < 0.05). Notably, in our cohort, there was no significant association between V-AKI and age, obesity, or baseline GFR. With regards to vancomycin-related variables (Table 3), receipt of higher doses, including doses > 4000 mg/day and loading doses, were not associated with an increased odds of V-AKI. Measurement of at least one vancomycin trough level was associated with an increased risk of V-AKI (*p* = 0.043). Although the number of patients with a trough > 20 mg/L was small (*n* = 25), supratherapeutic troughs were associated with increased V-AKI (OR 4.035, 95% CI 1.297–12.555). Longer durations of vancomycin therapy were associated with increased risk particularly among the approximately 20% of patients who received vancomycin for > 7 days (OR 2.364, 95% CI 1.171–4.775). The receipt of parenteral contrast dye or at least one concomitant nephrotoxic medication for ≥ 48 h was associated with a greater than two-fold increased odds of V-AKI (Table 3). The use of concomitant Gram-negative coverage (≥ 72 h) was associated with an even higher odds of V-AKI (OR 4.119, 95% CI 1.685–10.065).

Baseline variables independently associated with the occurrence of V-AKI in the multivariable logistic regression analysis were (Table 5): no medical insurance (aOR 3.451, 95% 1.310–9.090), chronic alcohol abuse (aOR 4.710, 95% CI 1.929–11.499) and ICU admission within the first 24 h of vancomycin therapy (aOR 4.398, 95% 1.676–11.541). After controlling for these variables, receipt of Gram-negative coverage (aOR 2.926, 95% CI 1.158–7.392) and vancomycin duration (aOR 1.143, 95% 1.037–1.260) were positive predictors of V-AKI.

**Table 5:** Final logistic regression model for the occurrence of vancomycin-associated acute kidney injury

Parameter	Adjusted odds ratio (95% confidence interval)	*p* value
No medical insurance	3.451 (1.310–9.090)	0.012
Chronic alcohol abuse	4.710 (1.929–11.499)	0.001
ICU admission within 24 h of vancomycin initiation	4.398 (1.676–11.541)	0.003
Receipt of Gram-negative coverage	2.926 (1.158–7.392)	0.023
Vancomycin duration^a^	1.143 (1.037–1.260)	0.007

*AUC* area under the curve, *ICU* intensive care unit

Overall *p *value (likelihood ration test) was < 0.001; Hosmer–Lemeshow test *p* = 0.340; AUC 0.776 (95% CI 0.700–0.851)

^a^The adjusted odds ratio for vancomycin duration reflects the increased likelihood of V-AKI for each one day increase in vancomycin length of therapy

The Cox proportional hazard model identified the following baseline variables significantly associated with V-AKI (Table 6): no medical insurance (aHR 2.898, 95% CI 1.303–6.445), chronic alcohol abuse (aHR 3.295, 95% CI 1.559–6.964), chronic pulmonary disease (aHR 2.151, 95% CI 1.027–4.503) and ICU admission within 24 h of vancomycin initiation (aHR 3.327, 95% CI 1.487–7.446). After controlling for these variables, the only treatment-related variable independently associated with V-AKI was receipt of concomitant Gram-negative coverage (aHR 2.812, 95% CI 1.160–6.817).

**Table 6:** Final Cox proportional hazards model for time to occurrence of vancomycin-associated acute kidney injury

Parameter	Adjusted hazard ratio (95% confidence interval)	*p* value
No medical insurance	2.898 (1.303–6.445)	0.009
Chronic alcohol abuse	3.295 (1.559–6.964)	0.002
Chronic pulmonary disease	2.151 (1.027–4.503)	0.042
ICU admission within 24 h of vancomycin initiation	3.327 (1.487–7.446)	0.003
Receipt of Gram-negative coverage	2.812 (1.160–6.817)	0.022

*ICU* intensive care unit

The final log likelihood ratio was–202.24. All covariates were dichotomized. The overall *p* value was < 0.001

We performed exploratory analyses to better understand the relationship between chronic alcohol abuse, no medical insurance and V-AKI. First, in patients with chronic alcohol abuse, the presence of most comorbid conditions was not higher with the exception of chronic liver disease (4.5 vs. 17.9%; *p* = 0.001) but there was no interaction between liver disease and alcohol abuse for V-AKI. Baseline serum creatinine, GFR and the presence of sepsis were similar. We did not discover differences in vancomycin dosing nor was there evidence of differential vancomycin exposure based on trough levels or vancomycin duration. We did however find that blood cultures were collected more often in these patients (68.9 vs. 87.2%; *p* = 0.017) and concomitant piperacillin-tazobactam was given to a higher proportion of patients with chronic alcohol abuse (18.4 vs. 35.9%; *p* = 0.009). Chronic alcohol abuse and concomitant piperacillin-tazobactam appeared to act synergistically to increase V-AKI risk: among patients who did not receive piperacillin-tazobactam, patients with chronic alcohol abuse was associated with an increased odds of V-AKI, although the association was not statistically significant (OR 2.733, 95% CI 0.856–8.731) but the association of chronic alcohol abuse and V-AKI was more pronounced in those who also received piperacillin-tazobactam (OR 5.00, 95% CI 1.405–17.793). Gram-negative bacteria were isolated in a similar proportion of patients without and with chronic alcohol abuse (15.9 vs. 10.5%; *p* = 0.743). Next, with regards to uninsured patients, when compared to patients with some form of medical insurance, uninsured patients were significantly younger [59 (47–70) years vs. 51 (39–60) year;, *p* = 0.027], had higher GFR by the Cockroft–Gault equation [95 (68–129) mL/min vs. 118 (89–154) mL/min;* p* = 0.016] and achieved lower vancomycin trough levels [12 (9–17) mg/L vs. 8 (6–10) mg/L; *p* < 0.001]. Conversely, uninsured patients were more likely to receive a concomitant nephrotoxic drug (59.5 vs 78.1%; *p* = 0.038), in particular piperacillin-tazobactam (16.4 vs. 62.5%;*p* < 0.001). However, the association between no medical insurance and V-AKI was qualitatively similar in those who did and did not receive a concomitant nephrotoxic drug (OR 3.466, 95% CI 1.309–9.177 and OR 3.063, 95% CI 0.328–28.577, respectively).

As shown in Table 1, rates of V-AKI varied widely across centers. Sensitivity analyses demonstrated that the final multivariable logistic regression model had similar discrimination and satisfactory calibration when patients enrolled from the Detroit Medical Center (*n* = 193) were excluded as well as when patients enrolled from sites that contributed less than ten cases were excluded (Supplementary Appendix 2).

With regards to other important patient outcomes, length of stay was significantly longer in patients who experienced V-AKI [9 (6–3) days vs. 6 (5–9) days; *p* = 0.001] and a significantly higher proportion of patients who experienced V-AKI were readmitted to the hospital within 30 days of discharge (30.8 vs. 9.0%; *p* < 0.001). Thirty-day all-cause mortality occurred in three patients: one (0.3%) in the non V-AKI group compared to two (5.1%) in the V-AKI group (*p* = 0.024).

## Discussion

Vancomycin is the most commonly prescribed antibiotic in US hospitals with recent studies finding approximately one in four patients receive vancomycin during the course of their hospital stay [49]. Among pharmacists, nephrologists and infectious diseases physicians, there is a broad range of beliefs about vancomycin’s propensity to cause V-AKI [50]. Previous studies have reported V-AKI rates ranging from less than 5% to greater than 40% and a wide variety of risk factors have been implicated [19, 33]. We conducted a focused analysis of this adverse effect specifically in hospitalized patients treated with vancomycin for ABSSSI. Our study sample was enriched with patients at higher risk of V-AKI because these patients have been poorly represented in recent randomized controlled ABSSSI studies. We believe our cohort is more broadly representative of real-world ABSSSI patients managed in the inpatient setting. We found that nearly one in ten patients experienced V-AKI. This represents a two- to three-fold increase over recent studies conducted for regulatory approval of new antibiotics that used vancomycin as the comparator and enrolled patients at low baseline risk [22–24]. Consistent with previous studies [19, 27], we found that patients who experienced V-AKI had significantly longer hospital lengths of stay, higher 30-day readmission rates and higher all-cause 30-day mortality. Although we are cautious not to ascribe causation, it is clear that patients who experience V-AKI place a significant burden on the healthcare system. In our study, the median length of stay of those who experienced V-AKI was 3 days longer than those who did not, which could translate into incremental costs to the hospital for room and board alone of approximately US$8000 depending on the geographic location and type of institution [51]. Additional costs are incurred for pharmacist’s time, laboratory monitoring, nephrology consult (required in 3.1% of our cohort) and dialysis (1.0% of our cohort). These findings underscore the need to better understand the individual patient- and drug-related risk factors that would enable clinicians to safely and effectively use vancomycin when it is indicated.

Some, but not all, previous studies have found increased rates of V-AKI with higher vancomycin trough levels [19, 27, 48]. In our cohort, higher initial troughs within the therapeutic range (< 20 mg/mL) were not positively associated with V-AKI. Supratherapeutic levels dramatically increased risk; however, few patients had trough levels above 20 mg/L resulting in imprecise estimates. Vancomycin is predominantly eliminated in the urine by glomerular filtration, therefore a decrease in renal function, from any etiology, will increase vancomycin serum concentrations [19, 50]. In addition, changes in serum creatinine are known to lag actual renal injury. This makes it difficult to establish an exposure–nephrotoxicity relationship. Aware of this, we restricted our analysis to initial trough levels which may explain the absence of an association for levels of 15–20 mg/L compared to other studies that have included levels collected throughout the course of therapy [19, 33].

Unlike previous studies, we also did not observe a positive relationship between vancomycin dose and V-AKI [33, 52]. However, less than 5% of patients received doses exceeding 4000 mg/day and the median daily dose (26 mg/kg) was less than the standard 30 mg/kg/day (15 mg/kg every 12 h). Loading doses were also uncommon (11.4%). The majority of our patients were obese. Other studies have found obesity to be a significant risk factor for V-AKI [18, 52]. Cognizant of this association, clinicians may have under-dosed heavier patients, minimizing our ability to detect a relationship between higher doses or obesity and V-AKI. The observation that patients who received vancomycin therapeutic drug monitoring (TDM) was associated with over a two-fold increased odds of V-AKI also suggests that clinicians were more inclined to monitor vancomycin therapy in individuals perceived to be at higher risk or perhaps when longer durations of therapy were planned. The fact that TDM did not entirely obviate this risk attests to limitations of trough level monitoring and underscores the need to minimize vancomycin use when safer alternatives are suitable, particularly in patients known to be at risk for V-AKI due to concomitant nephrotoxic medications and/or other risk factors.

Prior studies have found a significant association between longer vancomycin treatment durations and risk for developing V-AKI [18, 27, 53]. We found that each additional day of therapy beyond 72 h was independently associated with 14% increased odds of V-AKI, and that durations greater that 7 days conferred a greater than two-fold increased odds on univariate analysis. There is now overwhelming data supporting short-course therapy in patients with a variety of acute bacterial infections including hospitalized patients [54, 55]. Our study demonstrates the need to translate this data into current clinical practice to minimize iatrogenic harms of antibiotic therapy.

We found that novel risk factors, such as the absence of medical insurance and chronic alcohol abuse, were associated with V-AKI in our cohort. Liver disease was more prevalent among patients with chronic alcohol abuse and, even though baseline serum creatinine and GFR were similar in when stratified by chronic alcohol abuse and liver disease, patients with advanced liver disease may have renal impairment that is substantially more severe than suggested by serum creatinine concentration [56]. We found that concomitant nephrotoxic drugs, in particular piperacillin-tazobactam, were used more frequently in those with alcohol abuse and uninsured patients; however, this did not fully explain the increased V-AKI risk. This, coupled with the observation that blood cultures were collected more often in patients with chronic alcohol abuse, implies that the clinical picture may have suggested a more severe or complicated infection despite the fact that there was no difference in the objective signs we collected. These novel findings underscore the need to better characterize the epidemiology of ABSSSI in these vulnerable patient populations so that effective and safe management strategies can be developed.

One of the most important findings of this study was that IV vancomycin was not indicated in a large number of enrolled patients. Nearly 40% of patients had non-purulent cellulitis for which the predominant pathogen remains beta-hemolytic streptococcus, even in the post-CA-MRSA era [57, 58]. Narrow spectrum beta-lactams, which are not nephrotoxic (except for rare cases of interstitial nephritis) [59], are the drugs of choice for these infections [15]. In addition, over one-third of patients were candidates for upfront oral therapy based on meeting Dundee class I criteria. Provider perception that infections require IV antibiotics is a common reason for hospital admission in patients with ABSSSI. We now have a number of well-tolerated oral antibiotics with high oral bioavailability and appropriate spectrums of activity to treat ABSSSI [60–64]. Interventions targeted to stopping the continued overuse of parenteral antibiotics in the inpatient setting are needed.

Nearly 60% of patients in this study received combination therapy with a Gram-negative active antibiotic, yet only 16% of patients exhibited the high-risk features for which it is indicated (Dundee class IV). Exposing patients to antibiotics with an unnecessarily broad-spectrum of activity is unacceptable in the current era of escalating antimicrobial resistance and the increasing incidence and severity of *Clostridiodes difficile*-associated diarrhea [65]. In addition, receipt of Gram-negative coverage was strongly and independently associated with V-AKI. Mounting evidence suggests that the combination of vancomycin and piperacillin-tazobactam is associated with increases in serum creatinine [35, 66]. Although our sample size was not large enough to permit meaningful exploration, the risk in our study did not appear to be restricted to concomitant use of piperacillin-tazobactam only.

Our study has several important limitations. First, the study design was retrospective and observational and is therefore subject to the risk of bias inherent in this design. However, in-depth chart reviews allowed us to obtain detailed patient-level data and include variables that have not been assessed before. Next, we only enrolled patients treated with vancomycin and did not include a control group. As observed in this study, patients treated with vancomycin frequently have comorbidities and baseline organ impairment and are exposed to other agents which can be harmful to the kidneys, therefore we were not able to evaluate the attributable risk from vancomycin itself. However, our study reflects real-world practice since hospitalized patients often receive concomitant nephrotoxins and have multiple comorbidities. Our study was terminated early because of the sponsor’s financial status and we did not enroll our target sample size. Nearly half of the cohort were enrolled from one medical center, limiting generalizability. We observed wide ranges of V-AKI across sites with outliers often being sites that enrolled fewer patients. For these reasons, we conducted sensitivity analyses and found the results of multivariable analyses to be broadly consistent across sites. Antibiotic developers often fund valuable real-world research, and there is a desperate need for alternative payment models to sustain antibiotic development and related research.

## Conclusion

In conclusion, we found that nearly 10% of hospitalized ABSSSI patients treated with vancomycin experienced V-AKI. This adverse effect exerts a tremendous burden on the patients and the healthcare system and is largely preventable with three key changes in practice: (1) use oral antibiotics whenever appropriate, (2) treat non-purulent cellulitis with beta-lactams, and (3) when vancomycin must be used for ABSSSI, limit the duration of therapy to the absolute minimum.

## Electronic supplementary material

Below is the link to the electronic supplementary material.

Supplementary material 1 (DOCX 16 kb)
