# Supplementary material for: A Multicenter Evaluation of Vancomycin-Associated Acute Kidney Injury in Hospitalized Patients with Acute Bacterial Skin and Skin Structure Infections
Source: Infect Dis Ther. 2020 Jan 25;9(1):89–106. doi: 10.1007/s40121-019-00278-1 (PMC7054514; doi:10.1007/s40121-019-00278-1)
Supplement: Supplementary file 1 — Supplementary material 1 (DOCX 16 kb) [file 40121_2019_278_MOESM1_ESM.docx]

**Supplementary Appendix 1**

Risk factors for acute kidney injury

- Receipt of intravenous contrast dye
  - Concurrent use of nephrotoxic agent(s) for ≥ 48 hours (aminoglycoside, amphotericin B, colistin / polymyxin B, piperacillin-tazobactam, acyclovir, vasopressors, non-steroidal anti-inflammatories, ACE inhibitors, angiotensin receptor blockers, loop diuretics, and tenofovir)
  - Obesity (body mass index ≥ 30 kg/m^2^)
  - Creatinine clearance less than 50 mL/min as calculated by the Cockcroft-Gault equation ^36^
  - Age ≥ 65 years
  - Intensive care unit admission
  - Severe sepsis at initial presentation ^37^
  - Previous history of acute kidney injury
  - Diabetes with end organ damage
  - Heart failure Class III – IV
  - Cancer
  - Anemia

**Supplementary Appendix 2 – Sensitivity analysis**

| **Supplementary Table 1: Final logistic regression model for the occurrence of vancomycin-associated acute kidney injury excluding the Detroit Medical Center (n=222)^a^** | | |
| --- | --- | --- |
| Parameter | Adjusted odds ratio (95% confidence interval) | P value |
| No medical insurance | 3.965 (1.324 – 11.878) | 0.014 |
| Chronic alcohol abuse | 8.289 (2.636 – 26.070) | < 0.001 |
| ICU admission within 24 hours of vancomycin initiation | 3.220 (0.907 – 11.427) | 0.070 |
| Receipt of Gram-negative coverage | 2.579 (0.688 – 9.671) | 0.160 |
| Vancomycin duration^b^ | 1.220 (1.083 – 1.374) | 0.007 |
| ICU: intensive care unit   1. Overall P value (likelihood ration test) was < 0.001; Hosmer Lemeshow Test P = 0.099; AUC 0.823 (95% CI 0.736 – 0.909) 2. The adjusted odds ratio for vancomycin duration reflects the increased likelihood of V-AKI for each one day increase in vancomycin length of therapy | | |

| **Supplementary Table 2: Final logistic regression model for the occurrence of vancomycin-associated acute kidney injury excluding sites that contributed < 10 cases (n = 409)^a^** | | |
| --- | --- | --- |
| Parameter | Adjusted odds ratio (95% confidence interval) | P value |
| No medical insurance | 3.042 (1.070 – 8.654) | 0.037 |
| Chronic alcohol abuse | 3.636 (1.362 – 9.709) | 0.010 |
| ICU admission within 24 hours of vancomycin initiation | 4.725 (1.792 – 12.463) | 0.002 |
| Receipt of Gram-negative coverage | 2.537 (0.994 – 6.475) | 0.052 |
| Vancomycin duration^b^ | 1.164 (1.055 – 1.285) | 0.002 |
| ICU: intensive care unit   1. Overall P value (likelihood ration test) was < 0.001; Hosmer Lemeshow Test P = 0.726; AUC 0.770 (95% CI 0.691 – 0.849) 2. The adjusted odds ratio for vancomycin duration reflects the increased likelihood of V-AKI for each one day increase in vancomycin length of therapy | | |
